# Application of Heating on the Antioxidant and Antibacterial Properties of Malaysian and Australian Stingless Bee Honey

**DOI:** 10.3390/antibiotics10111365

**Published:** 2021-11-08

**Authors:** Nurul Ainaa Farhanah Mat Ramlan, Aina Syahirah Md Zin, Nur Fatihah Safari, Kim Wei Chan, Norhasnida Zawawi

**Affiliations:** 1 Functional Carbohydrates Research Laboratory, Faculty of Food Science and Technology, Universiti Putra Malaysia, Serdang 43400, Malaysia; n.ainaafarhanah@gmail.com (N.A.F.M.R.); ainamdz@gmail.com (A.S.M.Z.); nurfatihah1604@gmail.com (N.F.S.); 2 Natural Medicines and Products Research Laboratory, Institute of Bioscience, Universiti Putra Malaysia, Serdang 43400, Malaysia; chankim@upm.edu.my; 3 Laboratory of Halal Science, Halal Products Research Institute, Universiti Putra Malaysia, Serdang 43400, Malaysia

**Keywords:** heat treatment, stingless bee honey, antioxidant, antibacterial

## Abstract

In the honey industry, heat treatments are usually applied to maintain honey’s quality and shelf life. Heat treatment is used to avoid crystallisation and allow the easy use of honey, but treatment with heat might affect the antioxidant and antibacterial activities, which are the immediate health effects of honey. This study will determine the effect of heat treatment on Malaysian and Australian stingless bee honey (SBH) produced by the common bee species in both countries. Eighteen honey samples were subjected to heat at 45 °C, 55 °C and 65 °C for one hour and subsequently analysed for their total phenolic content (TPC), total flavonoid content (TFC), DPPH radical scavenging activity, ferric reducing antioxidant power (FRAP) and minimum inhibitory concentration (MIC). The results show that all samples had high TPC, TFC and antioxidant activities before the treatment. The heat treatments did not affect (*p* < 0.05) the TPC, TFC and antioxidant activities in most samples, but did inhibit the antibacterial activities consistently in most of the samples, regardless of the bee species and country of origin. This study also confirms a strong correlation between TPC and TFC with FRAP activities for the non-heated and heated honey samples (*p* < 0.05). Other heat-sensitive bioactive compounds in SBH should be measured to control the antibacterial properties present.

## 1. Introduction

Honey has significant components of sugar and water. Besides these, honey’s minor constituents include protein, phenolic acids, flavonoids, vitamins, enzymes, and minerals [1,2]. Stingless bee honey (SBH) differs from honey in taste, colour, and viscosity [3]. SBH has a unique sweet taste mixed with a sour and acidic taste. Besides its distinctive flavour, SBH consists of a phenolic and flavonoid profile that contributes to its good antioxidant, antibacterial, and anti-inflammatory properties [4,5]. However, each honey composition is different depending on the botanical origin, geographical region, and floral season, contributing to different strengths of antioxidant and antibacterial activities [6,7]. Honey’s antioxidant properties can be attributed to its phenolic and flavonoid compounds [8]. These phenolic and flavonoid compounds may also have antibacterial qualities, but other components such as hydrogen peroxide and non-peroxide components, such as its high sugar concentration and low pH, could have similar qualities [9]. In Malaysia, the most reared stingless bee species are *Heterotrigona itama* and *Geniotrigona thoracica*, while in Australia, *Tetragonula carbonaria* and *Tetragonula hockingsi* species are kept mainly by beekeepers in the state of Queensland [10,11].

Heat treatment has been introduced in honey processing due to the tendency of the honey to crystallise; heating prevents the crystallisation process from occurring and destroys microorganisms that can spoil the honey [12]. The liquefaction process at a temperature of 55 °C and the pasteurisation process are the two stages used in the honey industry to ensure that the honey remains in liquid form for an extended period, impairing the crystallisation nuclei [13]. However, a high temperature can affect the physicochemical properties of honey during processing [14], and this research will study the effect of heat treatments at temperatures of 45 °C, 55 °C, and 65 °C on the antioxidant and antibacterial activities of stingless bee honey from four different species and two different countries.

## 2. Results and Discussion

### 2.1. Total Phenolic Content

**Figure 1:**
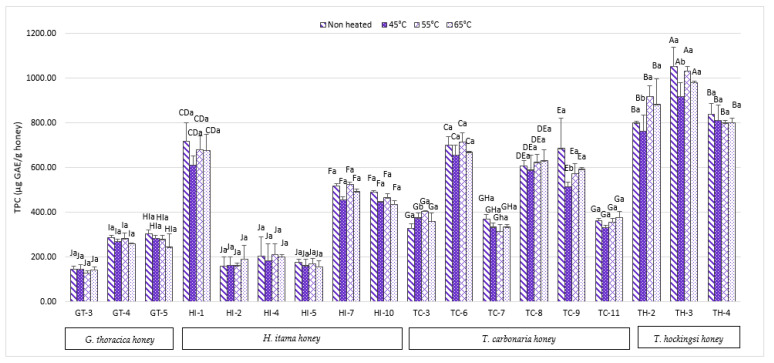
Total phenolic content of Malaysian and Australian stingless bee honey. The different superscripts, a and b, indicate significant differences (*p* < 0.05) between the temperatures, and the different superscripts A–J indicate significant differences (*p* < 0.05) between the stingless bee honeys.

Figure 1 shows the total phenolic content (TPC) of non-treated compared to heat-treated Malaysian and Australian SBH samples. The highest TPC was exhibited by non-treated Australian SBH from the *Tetragonula hockingsi* (TH-3) sample, with the value of 1053 ± 86.17 µg GAE/g. The highest TPC value found in the non-treated Malaysian SBH was from the species *Heterotrigona itama* (HI-1), with 717.37 ± 87.60 µg GAE/g. At 45 °C, the samples TC-9 and TH-2 had significantly (*p* > 0.05) reduced TPC. However, applying temperatures of 55 and 65 °C did not alter the TPC for any of the other samples. These findings did not agree with Jahan et al. [15], who reported an increased TPC content with increased temperatures in Bangladeshi honey samples treated under high temperatures of 50 °C, 70 °C and 90 °C. It is worth noting that Maillard Reaction Products (MRPs) might be produced at very high temperatures, contributing to a higher phenolic content [16]. According to a recent study by Majid et al. [17], stingless bee honey (*H. itama*) is abundant in the phenolic compounds chlorogenic acid (CLA) and p-coumaric acid. In contrast, a low amount was reported for epicatechin (EP), rutin (RE), catechin (CH), and protocatechuic acid (PTA). These phenolic compounds can act as antioxidants, and contribute to the TPC [3,9]. However, the phenolic compounds in stingless honey vary depending on the floral sources, season, and geographical area [17], as the phenolic compounds present in honey are closely related to the nectars, pollen, and floral species that the bee collected [18].

Another study by Braghini et al. [19] used a short heating period at high temperatures (90 °C and 95 °C, both for 15 s and 60 s) and reported increments of TPC after heat treatment. The individual phenolic compounds (p-coumaric acid, ferulic acid, chlorogenic acid, and protocatechuic acid) were found to be increased; a further investigation by Braghini et al. [20] using various heating temperatures and times displayed different findings. Fresh honey without heat treatment showed a high amount of p-coumaric acid (39.83 ± 0.82 µg/100 g), then after 60 min at 57 °C, the amount dropped to 22.27 ± 0.55 µg/100 g, and after 0.24 min at 70 °C, the amount increased to 38.83 ± 00.84 µg/100 g. In the same experiment, the vanillic acid content, which was at 30.82 ± 2.31 µg/100 g before heat treatment, increased to 94.57 ± 3.47 µg/100 g after 0.24 min at 70 °C. Hence, the TPC in heat-treated honey samples might fluctuate up to a specific temperature and was not negatively affected in the stingless honey samples, depending on the compounds.

### 2.2. Total Flavonoid Content

Flavonoids are low-molecular-weight phenolic compounds which are accountable for honey’s aroma and antioxidant potential [21]. Different floral sources and the bee species responsible for the honey production may contribute to different flavonoid types in the honey [22,23]. For instance, Sousa et al. [24] found that flavonoid rutin was high in stingless bee honey collected from *M. subnitida* and *M. scutellaris* species with botanical sources from botanical sources *Ziziphus Juazeiro*. However, another study by de Oliveira et al. [25] found that flavonoid rutin was absent in six stingless bee species, including *M. subnitida* and *M. scutellaris.* Majid et al. [17] discovered that rutin flavonoids were found only in one stingless bee honey of the *H. itama* species out of the six studied samples.

**Figure 2:**
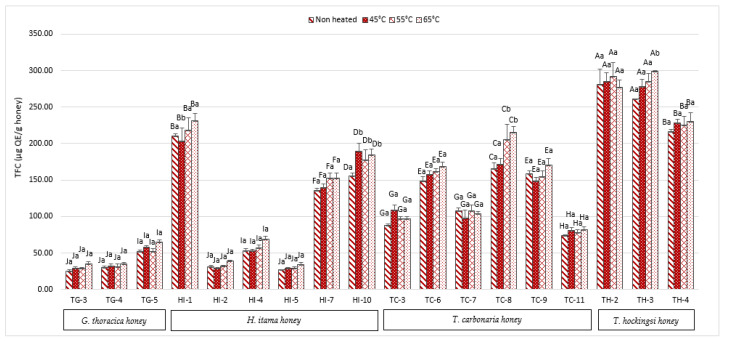
Total flavonoid content of Malaysian and Australian stingless bee honey. Different superscripts, a and b, indicate significant differences (*p* < 0.05) between the temperatures, and different superscripts, A–J, indicate significant differences (*p* < 0.05) between the stingless bee honeys.

Based on Figure 2, the highest TFC in heat-treated (65 °C) honey was found in the *T. hockingsi* sample, TH-3, at 299.06 ± 0.86 µg QE/100 g. There are no significant differences (*p* > 0.05) between the total flavonoid content (TFC) in the non-heat-treated and treated samples of Malaysian and Australian SBH, except for the samples HI-1, HI-10, TC-8, and TH-3. The TFC of these four samples increased significantly (*p* > 0.05) with increased temperatures, especially at 65 °C. In the study by Jahan et al. [15], the TFC also increased with increased temperatures of 50 °C, 70 °C, and 90 °C. The increment of the flavonoid content might occur because certain flavonoid compounds are activated due to the heat and increase to their bio-accessibility [15,26]. This finding agrees with Braghini et al.’s [20] research, which reported the appearance of the flavonoid compound, rutin, and isoquercetin in *Tetragonisca angustula* honey after being heated at a high temperature of 60 °C to 71 °C.

### 2.3. Inhibition of Free Radicals (DPPH) by Scavenging Activity

**Figure 3:**
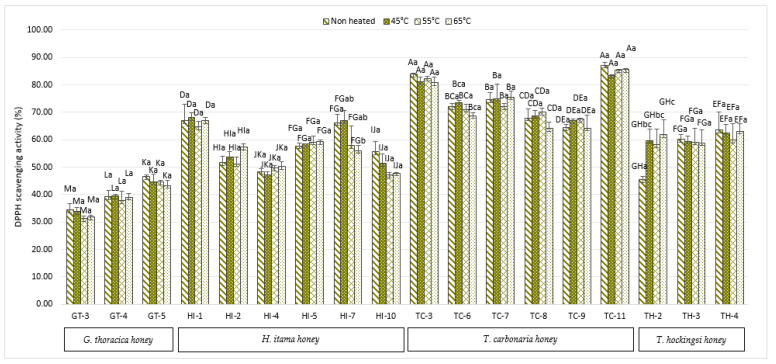
Percentage of the DPPH inhibition of Malaysian and Australian stingless bee honey. Different superscripts, a–c, indicate significant differences (*p* < 0.05) between the temperatures, and different superscripts, A–M, indicate significant differences (*p* < 0.05) between the stingless bee honeys.

DPPH scavenging activity inhibition can be measured by the ability of honey samples to reduce the DPPH free radical (purple colour) to diphenylpicrylhydrazine (yellow colour) [27]. Figure 3 shows the highest percentage of inhibition in the *T. carbonaria* sample (TC-11) at 87.15 ± 1.0%. No significant inhibition activity (*p* > 0.05) was found in any of the heat-treated Malaysian and Australian SBH samples, except for one sample of *H. itama* honey (HI-7) and one sample of *T. hockingsi* honey (TH-2). However, the effects were different because heating reduced the DPPH inhibition ability of the HI-7 sample but increased the ability in the TH-2 sample. A study by Turkmen et al. [16] found out that the heating of honey to 70 °C for ten days causes a notable increase in the percentage of antioxidant activities compared to 50 °C and 60 °C for 12 days. The time and temperature factors play essential roles in the antioxidant properties of the honey, as the process might induce the formation of brown pigments that can be recognised as MRPs, which have antioxidant properties, as reported in Amarowicz [28]. This study only conducted the heat treatment at 45 °C, 55 °C, and 65 °C for 1 h. From this, we can observe that the effect of the heat treatment was not significant compared to a previous study by Jahan et al. [15] and Turkmen et al. [16], as they used a high temperature and a longer heating time. Therefore, most of the results on the antioxidants show that there were no significant differences after the heat treatment.

The scavenging activity of stingless bee honey also depends on the flavonoid compounds, because the total number of hydroxyl groups, the configuration, and the substitution of flavonoids compounds affect their antioxidant activity [29]. A finding by Braghini et al. [19,20] shows that DPPH radical scavenging activity decreases at high temperatures. They reported that flavonoids such as chrysin and carnosol were high in fresh honey, at 21.1 ± 1.53 µg/100 g and 35.7 ± 0.96 µg/100 g, respectively. However, the amount reduces to the limit of quantitation (LOQ) after heating to 95 °C for 15 s and 60 s. The destruction of these flavonoid compounds might contribute to the decreasing value of the scavenging activity. Other flavonoids, such as quercetin and aromadendrin, were discovered to be increased as the heating temperature increased [20].

### 2.4. Ferric Reducing Antioxidant Power (FRAP)

**Figure 4:**
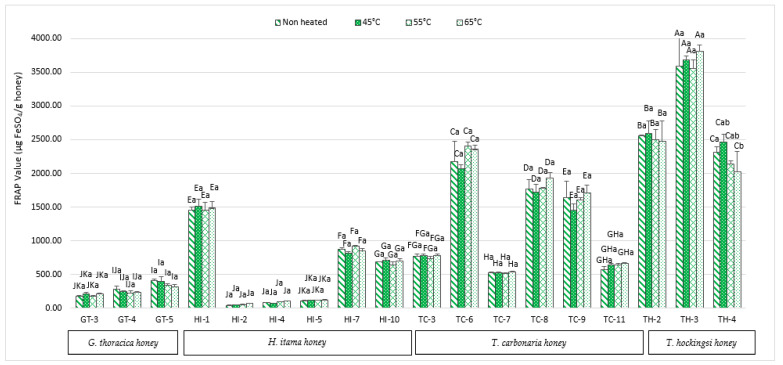
Ferric reducing antioxidant power value of Malaysian and Australian stingless bee honey. Different superscripts, a and b, indicate significant differences (*p* < 0.05) between the temperatures, and different superscripts, A–J, indicate significant differences (*p* < 0.05) between the stingless bee honeys.

The intense blue colour of a ferrous ion with a TPTZ complex occurs when high antioxidant SBH reacts with the pale yellow of a ferric ion with a TPTZ complex [30,31]. Figure 4 shows the results of the FRAP values for both Malaysian and Australian SBH. No significant differences (*p* > 0.05) were found in the non-treated and heat-treated SBH samples, except for Australian SBH from the species *T. hockingsi* (TH-4). The significant reduction of the FRAP value in the TH-4 sample can be seen at 65 °C. A low FRAP value can be seen in three Malaysian SBH from the species of *H. itama* (HI-2, HI-4, and HI-5) compared to the other Malaysian SBH. Low values of FRAP might be due to the low total phenolic content in the samples. As mentioned previously, honey possesses abundant phenolic compounds that can act as antioxidants [18]; thus, it works as a reducing agent in antioxidant activities.

The FRAP value in Bangladeshi honey increases as the temperature of the heat treatment increases [15]. This study applied high-temperature heat treatment at 50 °C, 70 °C, and 90 °C for a longer heating time (12 h), and significantly increased the FRAP value. In a study by Šarić et al. [32], a high temperature (95 °C) was applied for a shorter heating time (5 min), and the results for the FRAP value showed inconsistent changes in the antioxidant activity. Sixteen samples had lower FRAP values after heat treatment, while 14 samples had higher FRAP values. A study by Braghini et al. [19] displayed an increment of the FRAP value after the honey was treated at 90 °C and 95 °C for 15 s and 60 s, respectively. They also reported that some individual phenolic compounds like *p*-coumaric acid, ferulic acid, chlorogenic acid, and protocatechuic acid increase after heat treatment. This shows that those compounds might be the contributor to the increased antioxidant activity after heat treatment. Therefore, the heating temperature and time can affect the antioxidant activities in honey. As mentioned previously, the heat treatment used in this study was insufficient to activate the phenolic compounds and the production of MRPs, which might be one of the other sources contributing to the antioxidant activities.

### 2.5. Correlation between the Total Polyphenol and Total Flavonoid Content with the Antioxidant Activities

Table 1 showed the Pearson correlation (r) in non-heated and heated SBH. For both non-heated and heated honey, the TPC was found to have a strong relationship with the TFC and FRAP activity. TPC exhibits a strong correlation with TFC (r = 0.937) and TPC with FRAP activity (r = 0.952) in non-heated honey. TPC and TFC have an r = 0.942 correlation, TPC and FRAP activity have an r = 0.962 correlation at 45 °C, TPC and TFC have a r = 0.953 correlation, and TPC and FRAP activity have an r = 0.962 correlation at 55 °C. TPC activity had the greatest correlation with FRAP activity at both 45 °C and 55 °C, according to this data. TPC had the strongest correlation with TFC at 65 °C, with r = 0.956, while TPC has a strong correlation with FRAP activity at 65 °C, with r = 0.941. From these results, there were no differences in the correlation between non-heated and heated honey. These correlations indicate that the phenolic and flavonoid contents of all the honey can reduce the ferric ion activity. These can be seen in sample AA1, which has a high TPC (Figure 1), a high TFC (Figure 2), and an equivalent increase of FRAP activity (Figure 4). A study by Shamsudin et al. [33] also reported a similar finding, where a strong correlation coefficient (r) for TPC was shown with TFC (r = 0.802), as well as a strong correlation with FRAP activity (r = 0.981) when they tested stingless bee honey from species of *Heterotrigona itama* with different nectar sources, with no pre-treatment of the sample. In this study, heat treatments at up to 65 °C maintain the correlation between TPC and TFC, with antioxidant activity measured by FRAP but not DPPH.

### 2.6. Minimum Inhibitory Concentration (MIC)

**Figure 5:**
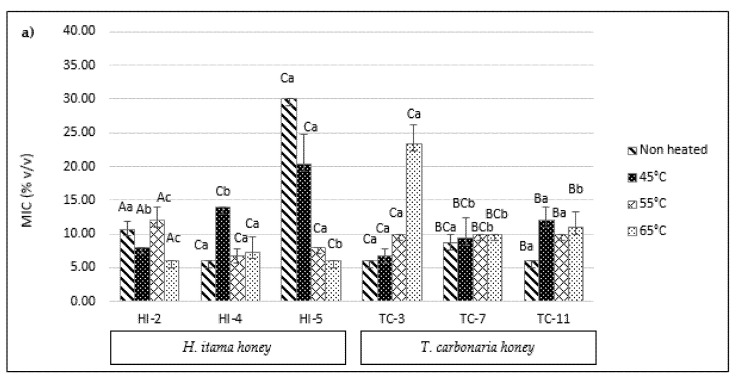

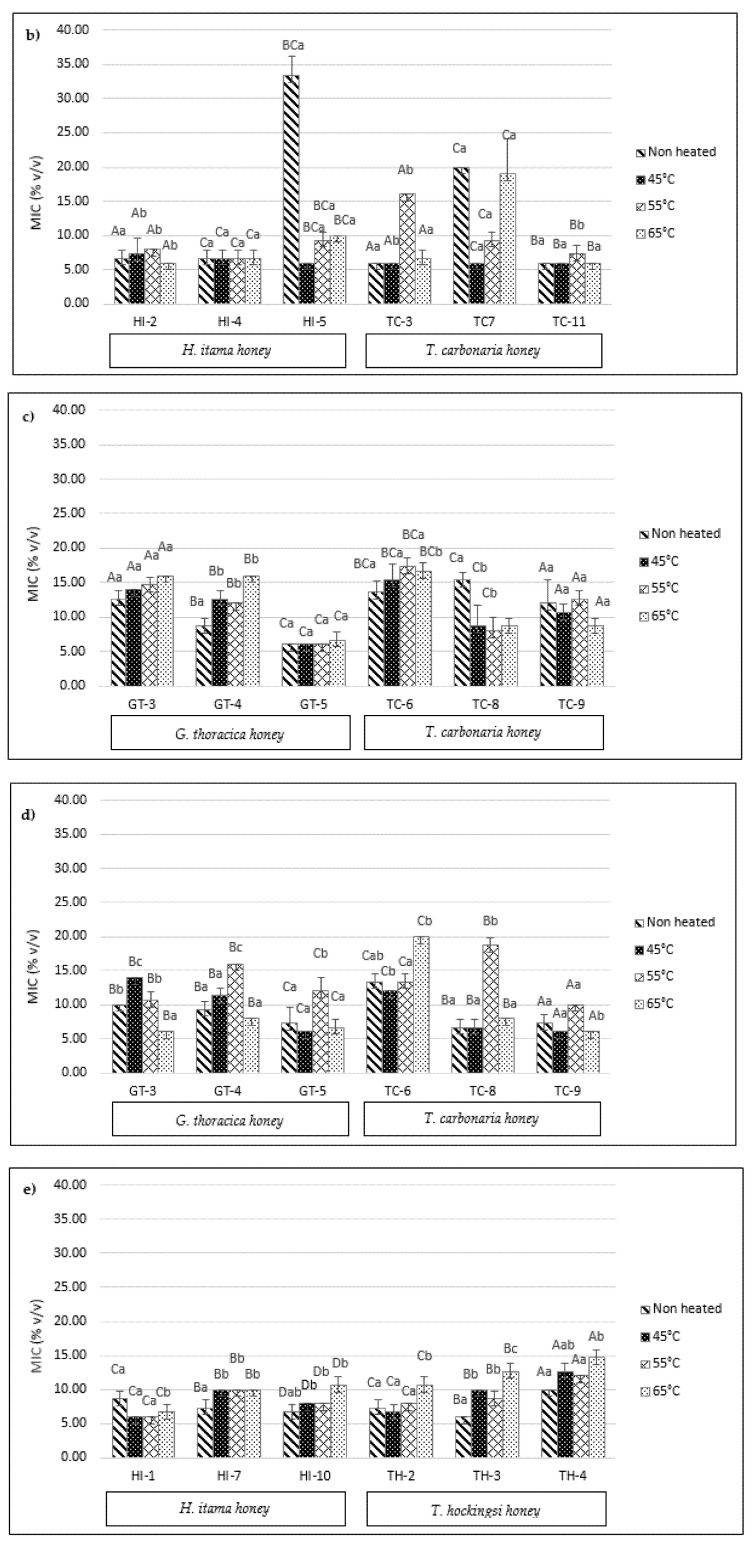

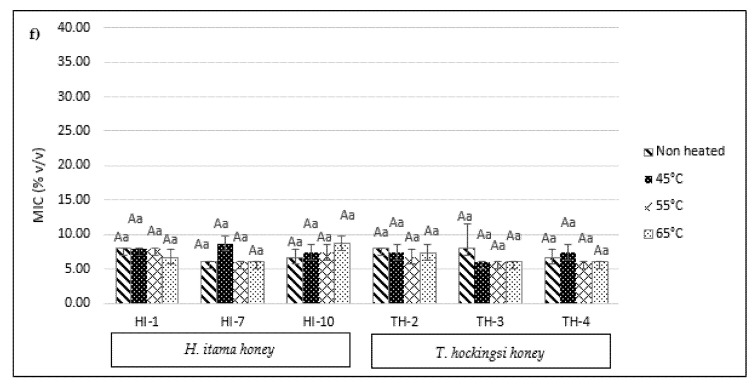
Minimum inhibitory concentration (MIC) of SBH from Malaysian and Australian against a few bacteria: (**a**) *Pseudomonas aeruginosa*, (**b**) *Escherichia coli*, (**c**) *Klebsiella pneumoniae*, (**d**) *Salmonella typhimurium*, (**e**) *Bacillus cereus*, and (**f**) *Staphylococcus aureus*. Different superscripts, a–c, indicate significant differences (*p* < 0.05) between the temperatures, and the different superscripts A–D indicate significant differences (*p* < 0.05) between the stingless bee honeys.

Every three different samples of SBH from Malaysia and Australia were tested against six different bacteria in these MIC assays. The MIC of Malaysian and Australian SBH against six distinct bacteria is shown in Figure 5. The minimum inhibitory concentration (MIC) is the lowest concentration of compounds required to stop bacteria from growing [34]. As a result, a lower MIC suggests more significant antibacterial activity. All of the SBH samples (non-treated and heat-treated) showed antibacterial activities within the range of 6% up to 34% SBH concentrations. In general, these results show that the Malaysian and Australian SBH have suitable antibacterial properties. According to Tuksitha et al. [5], the MIC values for non-heated stingless bee honey from *G. thoracica*, *H. itama*, and *H. erythrogastra* species against the bacteria *S. aureus* and *E. coli* were 5% *w*/*w*, 10% *w*/*w*, and 3% *w*/*w*, respectively. They also reported the same MIC values for *G. thoracica* and *H. itama* against *P. aeruginosa*, with 5% *w*/*w* and 10% *w*/*w*, respectively, whereas *H. erythrogastra* had an MIC value of 5% *w*/*w* against *P. aeruginosa.* Our findings demonstrate that two *H. itama* samples (HI-2 and HI5) for non-heated against P. aeruginosa had MIC values more significant than those found in earlier studies, which were 10.67% and 30% *v*/*v*, respectively. Nonetheless, compared to the previous experiment, two *H. itama* (HI-2 and HI-4) samples showed a lower MIC value of 6.67% *v/v* against *E. coli.* In comparison to Tuksitha et al. [5], *H. itama* against *S. aureus* had a lower MIC value (6% *v*/*v*–8% *v*/*v*) in this study. However, the heat treatments were found to negatively affect most of the SBH samples, especially at temperatures of 55 °C and 65 °C.

Figure 5a reveals SBH against the bacteria *P. aeruginosa*, with no significant differences (*p* > 0.05) for samples HI-2 and TC-7, but significant differences (*p* < 0.05) for the remaining samples after the heat treatments. As the temperature rose, sample HI-5 showed increased antibacterial activity (low MIC value). However, as the temperature rose, sample TC-3′s antibacterial activity decreased. There were significant differences (*p* < 0.05) in SBH (HI-5, TC-3 and TC-7) against *E. coli* bacteria (Figure 5b) after the heat treatment. At 45 °C to 65 °C, sample HI-5 showed a decrease in its MIC value, indicating a higher antibacterial activity, while sample TC-7 showed an increase in its MIC value as the temperature rose from 45 °C to 65 °C, indicating that it has reduced in antibacterial activity. Figure 5c shows SBH MIC against *K. pneumonia* bacteria, with significant differences (*p* < 0.05) between the Malaysian and Australian SBH samples. There were also significant differences in the MIC values with the temperature treatments (*p* > 0.05) for the *G. thoracica* (GT-4) and *T. carbonaria* (TC-8) samples. The antibacterial activity of sample GT-4 decreased as the temperature rose, but the antibacterial activity of sample TC-8 rose as the temperature rose. Figure 5d shows a significant difference (*p* < 0.05) in the SBH samples against *S. typhimurium* bacteria after the heat treatments. Starting at 55 °C, most of the samples (GT-4, GT-5, TC-8 and TC-9) decreased in their antibacterial activity. Heat-treated SBH samples against *B. cereus* bacteria result in significant differences (*p* < 0.05) (Figure 5e). As the temperature rose, the antibacterial activity of most of the samples against these bacteria decreased, except for samples from the species *H. itama* (HI-1). Our findings show that most SBH samples against bacteria *S. typhimurium* show decreased antibacterial activities starting at 55 °C.

Previous research which reported similar findings has been performed by Sulaiman and Sarbon [35], where they studied unheated and heated Malaysian SBH (the species name was not mentioned) at three different temperatures (50 °C, 70 °C, and 90 °C) treated against four specific bacteria: *Staphylococcus aureus*, *Escherichia coli*, *Salmonella*, and *Pseudomonas aeruginosa*. They discovered that untreated SBH against the four types of bacteria showed the lowest MIC value at 6.50%, correspondingly. As the temperature increases, the MIC value increases, indicating that the treated honey’s antibacterial activities have been reduced. Antibacterial compounds other than phenolic acids and flavonoids that could be affected by temperatures include hydrogen peroxide (H_2_O_2_). This is produced in the conversion process of glucose to gluconic acid in the enzyme glucose oxidase [36]. Chen et al. [37] wrote that heating honey for 20 min at 50 °C would affect the enzyme (glucose oxidase) and significantly reduce its enzyme activity. Certain flavonoids that are heat sensitive, such as quercetin and rutin, which also have antimicrobial compounds, could be affected by heat. These flavonoids were reported to have been utilised in MIC analysis against bacteria *E. coli* and *P. aeruginosa*, and both compounds exhibit moderate antibacterial activity (500 µg/mL) against both bacteria [38]. Other heat-sensitive flavonoids with antibacterial properties are apigenin, luteolin, and morin, which can inhibit *S. aureus* at doses of 1 mM [39]. The absence of these compounds may lead to lower antibacterial activities.

## 3. Materials and Methods

### 3.1. Honey Samples

Eighteen samples of unprocessed stingless bee honey were collected from hives located at different states in Malaysia and different cities in Queensland, Australia (see Table 2). None of the samples were subjected to any heating or filtration process. The samples were kept at 4 °C before analysis.

### 3.2. Bacteria Samples

The antibacterial activity of the honey samples was assessed against six bacterial strains: *Pseudomonas aeruginosa* ATCC 11,778, *Escherichia coli* TOP10, *Klebsiella pneumoniae* ATCC 13,883, *Salmonella typhimurium* ATCC 13311, *Bacillus cereus* ATCC 11,778, and *Staphylococcus aureus* ATCC 29,213, which were obtained from the Enzyme and Microbial Technology Research Centre (EMTech), Universiti Putra Malaysia (UPM).

### 3.3. Heating Procedure

The heat treatment was performed using the method developed by Bucekova et al. [40]. All of the honey samples were left at room temperature for two h before being subjected to the heating process at 45 °C, 55 °C, and 65 °C for 60 min using a water bath. Each of the honey samples was divided into 10 g and placed into 50 mL centrifuge tubes. The samples were heated using a conventional water bath (Protech 903, Balakong, Malaysia) for 30 min, and were then homogenised by turning the centrifuge tubes upside down for 5 min before heating for another 30 min. Untreated honey was used as a control.

### 3.4. Determination of the Total Phenolic Content

The total phenolic content was analysed by the Follin–Ciocalteu method, according to Hagr et al. [2]. A 100 μL diluted honey volume was mixed with a 500 μL Follin–Ciocalteu reagent, and then vortexed for 30 s. In total, 400 μL 7.5% (*w*/*v*) aqueous sodium carbonate was added, and vortexed again. The mixture was allowed to stand for 1 h, incubated at 40 °C in the dark. In total, 200 μL of the supernatant mixture was pipette out and loaded into a 96-well microplate. Methanol and distilled water acted as a blank. The absorbance of the reaction mixture was measured at 765 nm using a microplate spectrophotometer (Bio-Rad, Hercules, CA, USA). Gallic acid was used as a standard, with a concentration of 400 ppm, 100 ppm, 50 ppm, 25 ppm, 12.5 ppm, 6.25 ppm, and 3.125 ppm. The results were expressed as microgram Gallic acid equivalents per gram of honey (µg GAE/g honey).

### 3.5. Determination of the Total Flavonoid Content

The total flavonoid content (TFC) was determined by the aluminium colourimeteric method according to Hagr et al. [2] and Tuksitha et al. [5]. Quercetin was used as a standard, with concentrations of 100 ppm, 50 ppm, 25 ppm, 12.5 ppm, 6.25 ppm, 3.125 ppm, 1.56 ppm, and 0.78 ppm. In total, 100 μL of the diluted honey sample was mixed with 100 μL 2% aluminium chloride (AlCl_3_) in a 96-well plate. In total, 100 μL of each standard solution was combined with 2% AlCl_3_. In total, 100 μL distilled water was mixed with 100 μL 2% AlCl_3_, which acts as a negative control. In total, 200 μL ethanol was pipetted directly into a 96-well plate and served as a blank. The mixture was incubated for 10 min at room temperature. The absorbance of the mixture was measured at 450 nm using a Benchmark Plus Microplate (Bio-RAD170-6930, Singapore). The results were expressed as micrograms of quercetin equivalents (QE) per gram of honey (µg QE/g honey).

### 3.6. Determination of the Radical-Scavenging Effect on DPPH

The antioxidant activity of the honey was evaluated using a DPPH radical scavenging assay, as described by Hagr et al. [2]. The Trolox concentration of 100 ppm, 50 ppm, 25 ppm, 12.5 ppm, 6.25 ppm, 3.125 ppm, 1.56 ppm, and 0.78 ppm were used as a standard. In total, 50 μL diluted honey and standard Trolox was added to 195 μL DPPH reagent into a 96-well plate. In total, 50 μL methanol was also mixed with 195 μL DPPH reagent as a negative control. Those mixtures were incubated at room temperature in dark conditions and were allowed to stand for 1 h. The absorbance was measured at 540 nm using a microplate spectrophotometer (Bio-Rad, US), with methanol as a blank. The percentage of the radical scavenging activity was calculated as % Inhibition = [(blank absorbance—sample absorbance)/blank absorbance] × 100. The results were expressed as the % of DPPH inhibition.

### 3.7. Ferric Reducing Antioxidant Power (FRAP) Assay

The ferric reducing antioxidant power (FRAP) of each honey was analysed using the method from Hagr et al. [2] and Tuksitha et al. [5]. Ferrous sulfate (FeSO_4_) was used as a standard, with concentrations of 100 ppm, 50 ppm, 25 ppm, 12.5 ppm, 6.25 ppm, 3.125 ppm, 1.56 ppm, and 0.78 ppm. In total, 20 μL diluted honey was mixed with 220 μL FRAP reagent. The FRAP reagent was prepared by mixing 40 mL acetate buffer with 4 mL 2, 4, 6-Tri (2-pyridyl)-s-triazine (TPTZ) solution and 4 mL ferric chloride (FeCl_3_) solution. In total, 20 μL distilled water was mixed with the FRAP reagent, and acted as a negative control, while 240 μL methanol was a blank. The mixture was pipetted into the 96-well plate, and the absorbance of the mixture was measured at 593 nm using a Benchmark Plus Microplate (Bio-RAD170-6930, Singapore). The results were expressed as micrograms of FeSO_4_ equivalents per gram of honey (µg FeSO_4_/g honey).

### 3.8. Minimum Inhibitory Concentration (MIC) Analysis

The antibacterial activity of honey was evaluated using a minimum inhibitory concentration (MIC) assay, according to Bucekova et al. [40] and Tuksitha et al. [5]. The bacterial culture was inoculated in 10 mL Mueller Hinton Broth (MHB) and incubated at 37 °C for 16 h. In total, 100 μL bacterial suspension was inoculated with 4 mL phosphate-buffered saline (PBS) buffer, at pH 7.2. Then, the absorbance of the PBS buffer with the bacterial suspension was measured using a GENESYS 20 spectrophotometer (Thermo Fisher Scientific, Hercules, CA, USA) at 625 nm. The turbidity should be between 0.08–0.10. The mixture was diluted with PBS buffer if the turbidity was greater than 0.10 and was added with a bacterial suspension below 0.08. The serial dilution from 108 CFU mL^−1^ to 106 CFU mL^−1^ of the mixture was achieved by adding 1 mL of the mixture to 9 mL MHB. The dilutions of the honey samples were prepared at 50% (*v*/*v*), 45%, 35%, 30%, 25%, 20%, 18%, 16%, 14%, 12%, 10%, 8% and 6%. The total volume in the well should be 100 μL, with three replicates per dilution which each assay consist of (i) the test well: 90 μL of diluted honey was mixed with ten μL of bacterial suspension of 106 CFU mL^−1^; (ii) the dilution sterility: 100 μL honey dilution with MHB; (iii) the broth sterility control: 100 μL of MHB only (without honey and the bacterial suspension) that acted as negative control; and (iv) the viability control: 90 μL MHB with 10 μL bacterial suspension that acted as a positive control. The 96-well plate was then incubated for 18 h at 37 °C. The absorbance was measured at 490 nm using a Benchmark Plus Microplate (Bio-RAD170-6930, Singapore). The percentage inhibition of the bacteria growth for each honey dilution was calculated using the following formula:

(1) $$1-\frac{\left(Abs.\;of\;test\;well-Abs.\;of\;dilution\;sterility\;control\;well\right)}{\left(\;Abs.\;of\;assay\;viability\;control-Abs.\;of\;broth\;sterility\;control\right)}\times 100$$

The minimum percentage of the inhibition was 0%, and the maximum value was 100%.

### 3.9. Statistical Analysis

All of the analyses were conducted in triplicate, and the data are presented as the mean ± standard deviation. The statistical analyses were performed using Minitab 19.0 software, with an ANOVA one-way analysis of variance to determine the differences between the values of the tested samples and Tukey’s Test. The differences between the means using 95% confidence intervals (*p* < 0.05) were statistically significant. A correlation test was performed using Pearson correlation (r) at (*p* < 0.05), with Minitab 19.0 software, to evaluate the relationship between the antioxidants’ activities and the temperature.

## 4. Conclusions

In conclusion, heat treatments at temperatures of 45 °C, 55 °C, and 65 °C for 60 min have no effects on the TPC, TFC and antioxidant activities measured by the DPPH and FRAP values for most of the samples, regardless of the species and country of origin. The FRAP values were found to be correlated with TPC and TFC, but not the DPPH values. The phenolics and flavonoids with antioxidant properties are not the primary cause for antibacterial activities, because heating at set temperatures significantly reduced the antibacterial activities in most of the SBH samples from both countries. The different nectar sources and geographical locations of each honey sample may result in different individual TPC, TFC, antioxidant activities and antibacterial results. Various other bioactive compounds, such as organic acids or enzymes that are heat-sensitive, could contribute to the antibacterial effects in SBH. Thus, investigations of the stability of these compounds during heat treatments will give further explanations.

## Figures and Tables

**Table 1:** Pearson correlation of antioxidant activities for non-heated and the 45 °C, 55 °C and 65 °C heat treatment of SBH from Malaysia and Australia.

Antioxidant Analyses	TPC	TFC	DPPH	FRAP	TPC	TFC	DPPH	FRAP	TPC	TFC	DPPH	FRAP	TPC	TFC	DPPH	FRAP
	**RT**	**RT**	**RT**	**RT**	**45 °C**	**45 °C**	**45 °C**	**45 °C**	**55 °C**	**55 °C**	**55 °C**	**55 °C**	**65 °C**	**65 °C**	**65 °C**	**65 °C**
**TPC**																
*r*	-	0.937	0.233	0.952	-	0.942	0.359	0.962	-	0.953	0.313	0.962	-	0.956	0.321	0.941
*p*	-	0.000 *	0.091	0.000 *	-	0.000 *	0.008 *	0.000 *	-	0.000 *	0.021 *	0.000 *	-	0.000 *	0.018	0.000 *
**TFC**																
*r*	-	-	0.201	0.899	-	-	0.325	0.907	-	-	0.290	0.895	-	-	0.265	0.896
*p*	-	-	0.146	0.000 *	-	-	0.017 *	0.000 *	-	-	0.034	0.000 *	-	-	0.053	0.000 *
**DPPH**																
*r*	-	-	-	0.193	-	-	-	0.310	-	-	-	0.315	-	-	-	0.281
*p*	-	-	-	0.161	-	-	-	0.023 *	-	-	-	0.020	-	-	-	0.040 *

Asterisks (*) represent significant differences (*p* < 0.05). *r:* Pearson’s correlation coefficient; *p: p*-value. RT: room temperature.

**Table 2:** Stingless bee honey samples and their geographical origin.

No	Sample Code	Bee Species	Location
1	GT-3	*Geniotrigona thoracica*	Selangor, Malaysia
2	GT-4	*Geniotrigona thoracica*	Selangor, Malaysia
3	GT-5	*Geniotrigona thoracica*	Selangor, Malaysia
4	HI-1	*Heterotrigona itama*	Sarawak, Malaysia
5	HI-2	*Heterotrigona itama*	Selangor, Malaysia
6	HI-4	*Heterotrigona itama*	Selangor, Malaysia
7	HI-5	*Heterotrigona itama*	Johor, Malaysia
8	HI-7	*Heterotrigona itama*	Selangor, Malaysia
9	HI-10	*Heterotrigona itama*	Selangor, Malaysia
10	TC-3	*Tetragonula carbonaria*	Brisbane, Queensland, Australia
11	TC-6	*Tetragonula carbonaria*	Brisbane, Queensland, Australia
12	TC-7	*Tetragonula carbonaria*	Brisbane, Queensland, Australia
13	TC-8	*Tetragonula carbonaria*	Brisbane, Queensland, Australia
14	TC-9	*Tetragonula carbonaria*	Brisbane, Queensland, Australia
15	TC-11	*Tetragonula carbonaria*	Brisbane, Queensland, Australia
16	TH-2	*Tetragonula hockingsi*	Bargara, Queensland, Australia
17	TH-3	*Tetragonula hockingsi*	Bargara, Queensland, Australia
18	TH-4	*Tetragonula hockingsi*	Bargara, Queensland, Australia

## Data Availability

The data presented in this study are available.
